# Insights into the *Vibrio* Genus: A One Health Perspective from Host Adaptability and Antibiotic Resistance to In Silico Identification of Drug Targets

**DOI:** 10.3390/antibiotics11101399

**Published:** 2022-10-12

**Authors:** Pedro Henrique Marques, Lígia Carolina da Silva Prado, Andrei Giacchetto Felice, Thaís Cristina Vilela Rodrigues, Ulisses de Padua Pereira, Arun Kumar Jaiswal, Vasco Azevedo, Carlo José Freire Oliveira, Siomar Soares

**Affiliations:** 1 Department of Microbiology, Immunology and Parasitology, Federal University of Triângulo Mineiro, Uberaba 38025-180, MG, Brazil; 2 Interunit Bioinformatics Post-Graduate Program, Federal University of Minas Gerais, Belo Horizonte 31270-901, MG, Brazil; 3 Department of Genetics, Ecology and Evolution, Federal University of Minas Gerais, Belo Horizonte 31270-901, MG, Brazil; 4 Department of Preventive Veterinary Medicine, Londrina State University, Londrina 86057-970, PR, Brazil

**Keywords:** *Vibrio*, pan-resistome, one health, antibiotic resistance, subtractive genomics, virulence factors, genomic islands, molecular docking

## Abstract

The genus *Vibrio* comprises an important group of ubiquitous bacteria of marine systems with a high infectious capacity for humans and fish, which can lead to death or cause economic losses in aquaculture. However, little is known about the evolutionary process that led to the adaptation and colonization of humans and also about the consequences of the uncontrollable use of antibiotics in aquaculture. Here, comparative genomics analysis and functional gene annotation showed that the species more related to humans presented a significantly higher amount of proteins associated with colonization processes, such as transcriptional factors, signal transduction mechanisms, and iron uptake. In comparison, those aquaculture-associated species possess a much higher amount of resistance-associated genes, as with those of the tetracycline class. Finally, through subtractive genomics, we propose seven new drug targets such as: UMP Kinase, required to catalyze the phosphorylation of UMP into UDP, essential for the survival of bacteria of this genus; and, new natural molecules, which have demonstrated high affinity for the active sites of these targets. These data also suggest that the species most adaptable to fish and humans have a distinct natural evolution and probably undergo changes due to anthropogenic action in aquaculture or indiscriminate/irregular use of antibiotics.

## 1. Introduction

The *Vibrio* genus belongs to the family *Vibrionaceae* and is composed of Gram-negative bacteria, mostly flagellated, which live dispersed in aquatic environments, especially in marine waters, estuaries, and river mouths. They are usually concentrated in warm waters above 20 °C and develop better as the temperature increases [1,2]. It is common to identify the species associated with different organisms, such as fish, seabirds, algae, corals, crustaceans, mollusks, and mammals [3,4,5]. Likewise, there are pathogens from different hosts, such as human and fish pathogens, which stand out for causing economic damage to aquaculture and public health each year.

In humans, the pathogenic species of the *Vibrio* genus cause two diseases: cholera and vibriosis. In fish, on the other hand, a more significant number of the *Vibrio* species cause several types of vibriosis, such as warm water vibriosis by *V. vulnificus* biotype 2. Moreover, some opportunistic species from the genus are recognized as pathogens of fish and other animals, such as *V. harveyi* [6,7]. However, on rare occasions, opportunistic species may infect humans after a shark bite or boat propeller injury, for instance, with symptoms ranging from sepsis to limb infections [8,9]. Currently, new studies concluded that despite the few cases related to *Vibrio*-non-cholera, a worldwide increase in reports of vibriosis [10] might be expected soon.

Using epidemiological data available from the two CDC (US Centers for Disease Control and Prevention) monitoring tools, FoodNet and CoVis (Cholera and Other *Vibrio* Illness Surveillance), a comparative study of the infectivity and mortality of the *Vibrio* genus was performed in the two databases [11]. Based on these data, it is possible to differentiate the species of the genus (Supplementary Table S4) into pathogens that are commonly infectious to humans, with more than 30 cases per year (these are: *Vibrio alginolyticus, Vibrio cholerae, Vibrio fluvialis, Vibrio mimicus, Vibrio parahaemolyticus, Vibrio vulnificus*) [12,13,14,15,16,17], from those that rarely infect, with no more than 5 cases per year (these are: *Vibrio campbellii, Vibrio cincinnatiensis, Vibrio furnissii, Vibrio harveyi, Vibrio metoecus, Vibrio metschnikovii, Vibrio navarrensis*) [16,18,19]. Besides the impact on human health, *Vibrio alginolyticus, Vibrio harveyi, Vibrio anguillarum, Vibrio campbellii, Vibrio furnissii, Vibrio parahaemolyticus, Vibrio rotiferianus, Vibrio vulnificus* BT2 are the main fish pathogens [20,21,22,23,24,25,26]. However, the reasons why these different species have evolved to the point where they infect different organisms or can be asymptomatic or provoke disease, to a different extent, remains to be entirely elucidated.

In general terms, the pathogenicity of a microorganism, such as those from the *Vibrio* genus, is reflected by its ability to develop host adaptation, sometimes by acquiring novel virulence factors, such as through pathogenicity islands (PAIs), a class of genomic islands. Thus, it is expected to find differences between species that are best known as human pathogens and species that are pathogenic to fish, and of course, differences in species that rarely infect either organism [27,28]. Therefore, the analysis of genomic plasticity, together with the understanding of these differences and the prediction of the ability of species to modify their genomes and evolve by leaps in the future by acquiring factors that will culminate in the ability to infect other organisms is an essential strategy to control the spread of these bacterial pathogens [29,30]. Genomic islands such as PAIs can be determinants for pathogenicity in *Vibrio* spp. As verified in *V. cholerae,* the VPI-1 encodes virulence factors crucial for intestinal colonization, facilitating the development of cholera [31].

Another important factor when analyzing genomic differences in microorganisms is antibiotic resistance. In aquaculture, drugs can be administered directly into the water or food, such as tetracycline, a drug widely used in this environment. Nevertheless, exposure to constant concentrations of antibiotics for an extended period may cause selective pressure on microorganisms, contributing to the development and spread of resistant bacteria and ARGs (antibiotic resistance genes) [32]. In *Vibrio* spp., numerous studies report resistant strains of species such as *Vibrio parahaemolyticus* and *Vibrio vulnificus* [33,34]. Inevitably, this interferes with human infections, as the bacteria share the environment with fish. With the resistance acquisition, the bacterium may, through ingestion of the contaminated fish, infect and worsen the clinical condition of patients [35]. Furthermore, the trend of antibacterial resistance is increasing, as previous studies have shown that global warming and improper disposal of plastic in the oceans are providing a suitable environment for bacterial growth and, consequently, increased biofilm formation and horizontal gene transfer (HGT) rates, including ARGs [36,37,38]. These data, taken together, indicate that several factors are involved in the infective capacity, pathogenicity, and adaptation of different species of bacteria of the *Vibrio* genus.

Currently, antibiotic resistance is a serious global public health problem, which is mainly noticed at the hospital level, during the treatment of infected patients. However, for *Vibrio* spp. part of this problem must be traced back to the aquatic environment and intensive aquaculture [39]. In this sense, we approach the “One Health” perspective from the bioinformatics point of view to better understand the differences between species of the genus *Vibrio* and genetically describe, as far as possible, those with the most significant potential for adaptation, pathogenicity, and the ability to resist antibiotics. In other words, we verify antibiotic resistance profiles and their correlation with the impact on humans and aquaculture. Moreover, we used an in silico approach to identify drug candidates for bacteria of the *Vibrio* genus that affect only humans or fish or both hosts. This choice was made because humans and fish are the most epidemiologically and economically relevant hosts.

## 2. Results

### 2.1. Human Pathogens Have More Genes Associated with Host Adaptation Than Fish Pathogens

**Figure 1:**
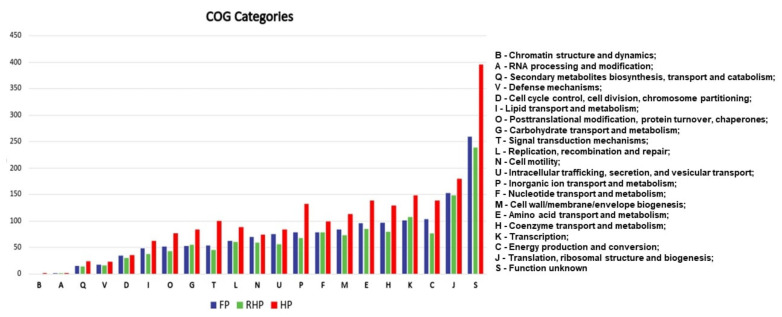
COG classifications made by eggNOG-mapper. Each category is represented by a letter, and each column corresponds to a group of pathogens. The HP group is red, green RHP, and blue FP.

By clustering orthologous genes with the OrthoFinder tool, we could identify the core genomes of each of the three groups. In the Human Pathogens group (HP), we predicted a core genome corresponding to 2054 proteins. In the Fish Pathogens group (FP), we found 1461 corresponding proteins, and in the Rare Human Pathogens group (RHP), 1330. These proteins were functionally classified by eggNOG-mapper into 21 COG categories (Supplementary Table S1), where each letter corresponds to a category (Figure 1).

Among the 21 categories, we observed that, in general, the HP group has a more significant number of conserved proteins, with emphasis on some in which there is a much larger number of proteins involved than in the other two groups. This phenomenon occurs mainly in categories T, P, E, H, and K (“Mechanisms of signal transduction”, “Transport and metabolism of inorganic ions”, “Transport and metabolism of amino acids”, “Transport and metabolism of coenzymes” and “Transcriptional”, respectively) where there are about 50 more proteins than in the other groups. Within category K of transcriptional processes, the P2RP tool classified the 148 proteins from HP, 107 from RHP, and 101 from FP into the categories: “Response Regulators”, “Transcriptional Regulators”, “One Component Systems”, and “Sigma Factors” with some proteins not fitting into any of the classifications (Table 1).

From the proteins classified in the P category (Transport and metabolism of inorganic ions), we could identify that, from 132 HP proteins, at least 21 had functional annotations associated with iron uptake, transport, and metabolism. Using the GIPSy tool, we identified pathogenicity islands (PAIs), metabolism islands (MIs), and resistance islands (RIs) in the reference genome of *Vibrio parahaemolyticus* strain RIMD 2210633. We found eleven PAIs on chromosome 1 and nine on chromosome 2. In the second PAI of chromosome 2 (coordinate: 414077…458149), it is visible that four proteins (Supplementary Table S1) involved in iron metabolism are present (Table 2). Information such as genes and biological activities of the proteins was retrieved from UniProt (Table 3).

**Figure 2:**
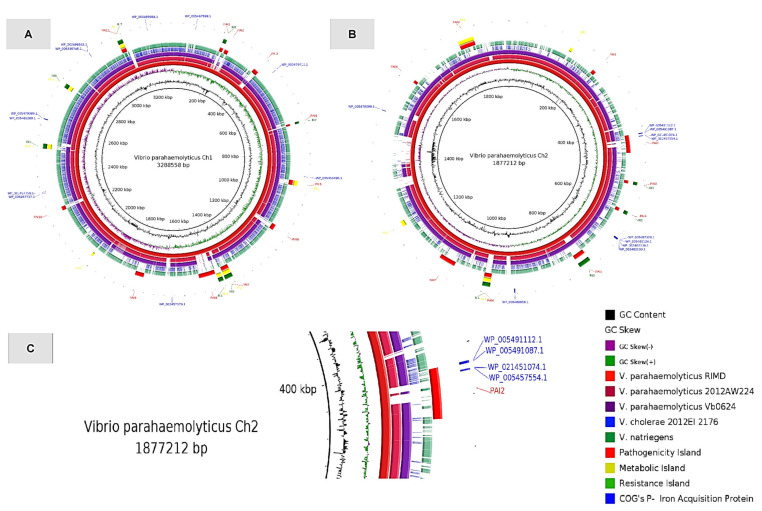
Genomic islands present in the genome of *Vibrio parahaemolyticus*. The BRIG analysis for the two *Vibrio* spp. chromosomes (Ch1-**A**; Ch2-**B**). In the order: *Vibrio parahaemolyticus*, strains RIMD, Vb0624, and 2012AW224, *Vibrio cholerae* strain 2012El 2176, *Vibrio natriegens*, strain COUG 16373. On the outer ring are plotted the genomic islands. In red are the pathogenicity islands, in green are the resistance islands, and in yellow are the metabolic islands predicted by GIPSy. In blue, there are small, enumerated traces, which correspond to the position of the iron metabolism proteins. **C**-Highlighted is the region of the proteins present in PAI2, in order: *WP_005491112.1, WP_005491087.1, WP_021451074.1* and *WP_005457554.1*.

The results of the genomic islands analyzed can be visualized in the BRIG tool, which plots a comparison using rings, where each ring corresponds to a genome (Figure 2). We used the *V. parahaemolyticus* RIMD2210633 genome as a reference since the islands were predicted for this strain. In sequence, we inserted two genomes from the same species, the 2012El 2176 genome of *V. cholerae* from the Haiti cholera outbreak as a pathogenic reference and *Vibrio natriegens* as a non-pathogenic reference. More externally, there is the prediction of genomic islands, with PAI2 of chromosome 2 highlighted in red and four blue dots plotted showing proteins involved in iron metabolism.

### 2.2. Bacteria of the Vibrio Genus Associated with Fish Infection Have a Much Higher Amount of Resistance-Associated Genes

**Figure 3:**
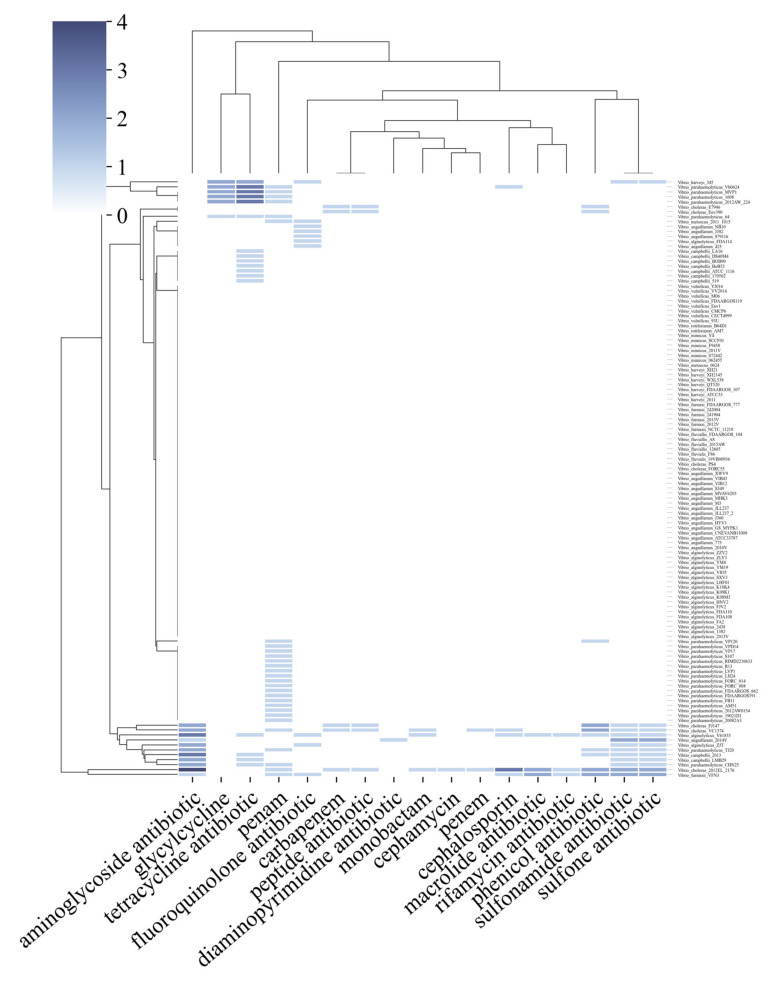
Clustering resistance genes from the PRAP tool using the CARD database. On the right are the genomes of each species used, below are the antibiotic classes, and on the left and above are the cluster formation process based on antibiotic resistance. From 0 to 4 indicates the number of genes associated with the antibiotic classes.

The first point to highlight from the data obtained from the PRAP tool is that out of 12 species analyzed, 8 harbor ARGs to at least one class of antibiotic (corresponding to more than 60% of the species). Some classes with the highest amounts of ARGs were aminoglycosides, tetracyclines, the b-lactams such as penems and carbapenems, and sulfonamides (Figure 3). The species that presented the most ARGs were *V. cholerae*, *V. parahaemolyticus, V. alginolyticus,* and *V. campbellii*. Further analysis could be conducted from specific isolates (Supplementary Table S2).

**Figure 4:**
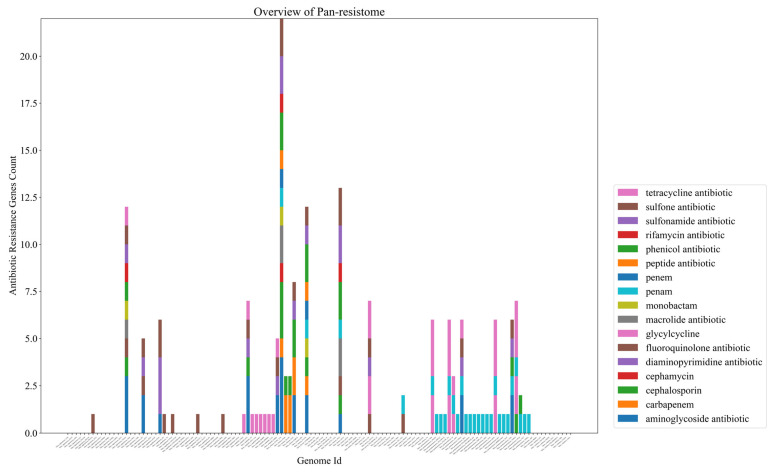
An overview of the pan-resistome of the *Vibrio* genus. Each bar per column corresponds to an ARG of a given genome. Each color represents resistance to a class of antibiotics, such as pink to tetracycline and light green to cephalosporin. For more details, check the chart in Supplementary Material 2.

The 2012El-2176 strain of *V. cholerae* isolated from an outbreak in Haiti had the most ARGs in its genome, with more than 10. Another fact is that four recent isolates from China have different resistance patterns than the others. *V. furnissii strain* VFN3, *V. alginolyticus* strain Vb 1833, *V. parahaemolyticus* strain Vb0624, and *V. campbellii* 20130629003S01 have, after *V. cholerae*, the highest amounts of ARGs, and the first three were isolated near Macau, in fish markets, or near civilization (Figure 4).

**Figure 5:**
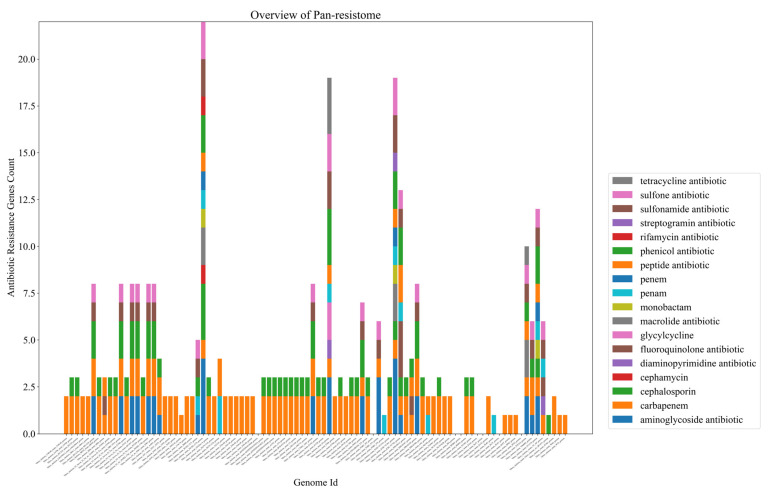
An overview of the pan-resistome of *Vibrio cholerae*. A total of 92 complete genomes were used for ARG prediction.

**Figure 6:**
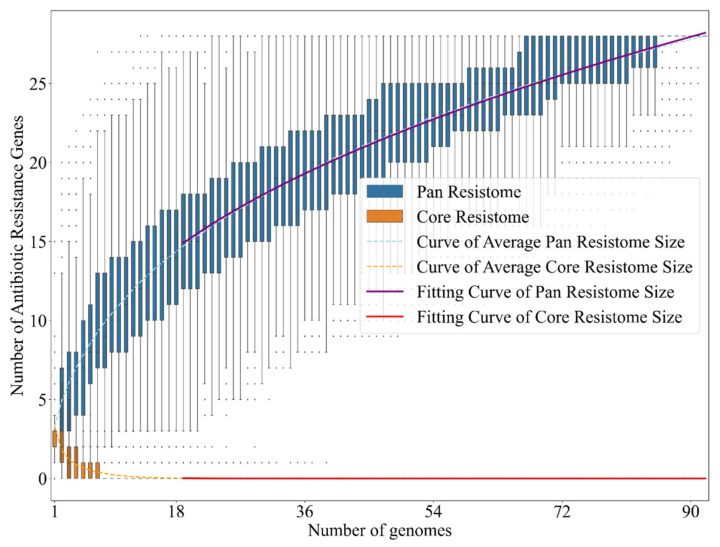
Development of the pan-resistome of *Vibrio cholerae*. Used the power law and combinatorial analysis to predict the addition of new ARGs per genome in the species. The purple line comprises the fitted curve of the pan-resistome appropriate for its development, while the bars in blue represent the addition of new ARGs. The same for the core-resistome in orange.

Furthermore, another influential factor is that all nine complete *V. campbellii* genomes available at NCBI have at least one tetracycline resistance gene from class “tet(35)”. To conclude, we obtained, with PRAP, the pan-resistome development of over 90 complete *V. cholerae* genomes given by the formula: “*p* = (4.527) × x^(0.404)(R^2 = 0.999)”, and the core-resistome, given by the formula: C = (4204559.706) × x^(−6.561)(R^2 = 0.989). Overall, almost all genomes analyzed in *V. cholerae* possess some ARG, such as cephalosporin class and others, and the pan-resistome development curve continues to rise without showing stabilization (Figure 5 and Figure 6).

### 2.3. Subtractive Genomics Reveals Seven Potential Drug Targets to Fight against Bacteria of the Vibrio Genus

After subtractive genomics, the filtering of proteins by cytoplasmic subcellular localization in the SurfG+ tool generated 309 cytoplasmic proteins for HP and 92 for FP. After that, we modeled all of them by homology with MHOLline, identifying 15 with very high structural quality for HP, 22 for FP, 6 with very high quality, and 16 with high quality. In addition, using DEG, only those considered essential, with a score lower than an e-value of 10 × 10^−6^, were used in the MHOLline tool, thus identifying three drug targets for HP and four targets for FP (only one with very high quality), being one of them common to both groups. The proteins predicted as suitable drug targets, their structural quality, molecular weights, metabolic pathways, and their respective genes are shown below (Table 4).

**Figure 7:**
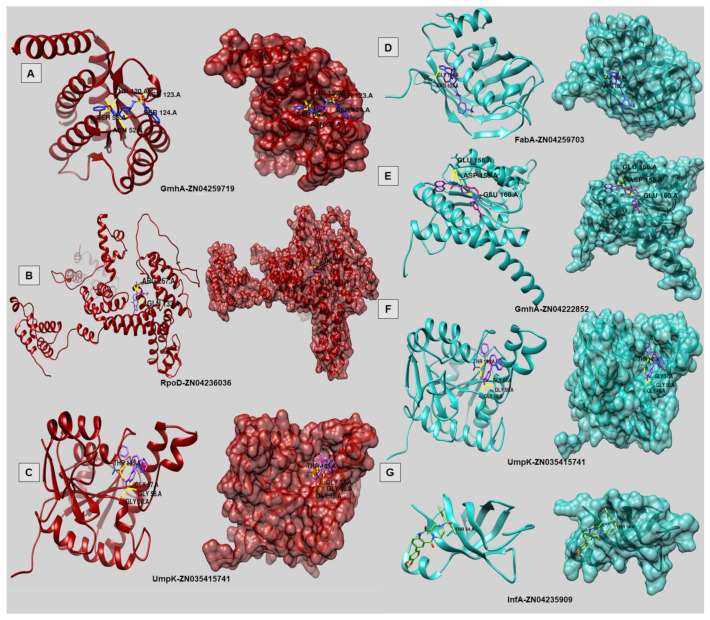
Docking results for HP and FP. (**A**–**C**) (Red) correspond to the results of HP docking, and (**D**–**G**) (Blue) correspond to the result of the second docking of the FP group. These results contain the respective drug targets with the best interactions with ZINC compounds. On the left are ribbons on the one, and on the right, the protein has its surface.

After preparing the receptors and forming two distinct ligand libraries: 5008 compounds for HP and 2202 for FP (Supplementary Table S3), docking was performed with AutoDockVina. After the docking analysis, we used a python script to identify the top molecule. Later, the top 10 molecules of each identified drug target for both HP and FP groups were analyzed individually for their binding energy. For the HP drug targets, UmpK, GmhA, and RpoD, compounds “ZN035415741”, “ZN04259719,” and “ZN04236036” showed high affinity, respectively, and for FP, UmpK, GmhA, FabA, and InfA bound strongly to “ZN035415741”, “ZN04222852”, “ZN04259703” and “ZN04235909”. In all cases, hydrogen bonds were present, mainly in residues of the active sites (Table 5). The images were extracted with Chimera Visualization Tool (Figure 7).

Briefly, human pathogens have more genes in common, classified by COG as part of the machinery responsible for ion, amino acid, and coenzyme maintenance. They also have orthologous signal transduction mechanisms and transcriptional proteins (from transcriptional factors and sigma factors to one-component systems), which is not the case in fish pathogens or other figural species of the genus. On the other hand, fish pathogens represent the species with the highest resistance to antibiotics such as tetracycline. Moreover, among them, Chinese isolates associated with aquaculture stand out, besides *V. cholerae,* some of the only ones to show resistance to multiple antibiotics. Through subtractive genomics seven proteins, four from fish pathogens and three from human pathogens, were discovered as drug targets. All have strategic intracellular localization, have no host homologs, and are considered essential for bacterial life. Thus, as a means to combat such pathogens, new natural compounds selected from virtual screening possess the ability to bind to the active site of the proteins, i.e., to the key amino acids critical for protein function.

About identified drug targets: IF-1 translation initiation factor (InfA) in FP is an essential protein for initiating protein synthesis, stabilizing the binding of IF-2 and IF-3 in the 30 S ribosomal subunit. Thus, helping to modulate mRNA selection by producing the 30 S pre-initiation complex (PIC) for protein synthesis. GmhA (Phosphoheptose isomerase) in both human and fish pathogens is involved in carbohydrate biosynthesis, a fundamental part of bacterial survival. The enzyme FabA (bifunctional 3-hydroxydecanoyl-ACP dehydratase/trans-2-decenoyl-ACP isomerase) in FP is necessary to introduce cis unsaturation in fatty acids, directly involved in fatty acid biosynthesis and lipid metabolism. Finally, the RpoD protein in HP (RNA polymerase sigma factor) is a sigma factor required to promote the binding of RNA polymerase to specific initiation sites. This sigma factor is the primary sigma factor during exponential growth. The Ump Kinase protein is an essential in vivo survival factor [40] catalyzing UMP phosphorylation.

## 3. Discussion

Cholera is one of the deadliest diseases of all time and is still a cause for concern today. Likewise, only the *Vibrio* species *V. vulnificus* is estimated to be responsible for more than 95% of seafood-associated deaths in the United States [41]. Underwater, *Vibrio* spp. is a nightmare for fish farming, mainly affecting the economy of Asian countries, as generating an estimated loss of 7% of the price of a kilogram of seabass on the East Coast of Peninsular Malaysia [42] among other many losses. Therefore, understanding the problem and discussing solutions is essential for human and veterinary medicine and the world economy. Our findings indicate that between these two major problems, the pathogens of the *Vibrio* genus differentiated themselves along the evolutionary process. In the first case, maintaining a much more considerable amount of proteins essential for the colonization of the human host, from transcriptional factors to signal transduction mechanisms, would allow such pathogens to adapt to changes in external physicochemical parameters. In addition to enhanced host survival, analyses of the genomic plasticity of these microorganisms revealed pathogenicity islands containing genes essential for iron uptake, which are crucial for the metabolism and pathogenicity of these species. In the second case, genomic analysis supports the theory that the persistence of vibriosis in the aquaculture environment and the fish hosts is shown to be conditioned by increasing resistance to the primary drugs used in the marine environment by the probable dissemination of ARGs. To this end, we identify seven new intracellular targets and propose new natural molecules with high binding capacity and affinity for the active site of these targets.

Among the conserved factors in the HP group is the transport and metabolism of amino acids and coenzymes, which are essential for bacterial physiological functioning in different environments [43]. Moreover, transcriptional proteins and signal transduction mechanisms are highly conserved in human pathogens; this fully reflects how bacteria can adapt to different environments, such as saline water and the human body. Changes in physicochemical parameters such as pH, osmolarity, temperature, and salinity can quickly kill unprepared microorganisms [44,45,46]. However, perceiving such changes through stimuli (such as the presence of bile in the human gastrointestinal tract) is one of the main obstacles to be overcome by some species by altering their gene expression, for example, by reallocating outer membrane proteins, thus allowing survival in different acidic concentrations [47]. Thus, the two moments are highly relevant: the perception of the external environment by transcriptional factors and the transduction of signals, so there is an alteration in gene expression and adaptation to the host.

Recently a question has been raised, how do waterborne pathogens such as *Vibrio* and Aeromonas species regulate gene expression to adapt to distinct hosts [28]. Through our results, we argue that transcriptional factors such as one-component systems, sigma factors, and response regulators are evolutionarily conserved in human pathogens and are one way to understand how transcriptional proteins affect host–pathogen interaction.

Finally, proteins involved in the processes of uptake, transport, and metabolism of inorganic ions are critical for adaptation as means of expelling and regulating ionic concentrations, such as sodium, which in the marine environment is highly concentrated, unlike the GI tract [48,49]. The proteins involved in the acquisition, metabolism, and transport of iron stand out among many inorganic ions. The *Vibrio* genus generally requires iron for growth. Iron in its two states (Fe^3+^ and Fe^2+^) can participate in electron transfer over a wide range of redox potentials and is essential for the functionality of various enzymes. For example, in the process of DNA synthesis and energy production. In these enzymes, the iron cofactor can be heme, an iron-sulfur cluster, or, less frequently, an iron atom [50].

By analyzing genomic islands of *V. parahaemolyticus*, a known human pathogen, we were able to identify that in at least one pathogenicity island, there is the presence of four proteins involved in the uptake and transport of the heme group. For example, the protein HutZ (WP_005457554.1) is critically important for the life of *V. cholerae* and may act in the storage of the heme group [51]. The protein HutX (WP_021451074.1) acts with HutZ in the transport and storage of the heme group and is more precisely involved in the intracellular traffic to bring the heme group to HutZ [52].

Similarly, the two other proteins are involved between external heme uptake and delivery to carrier proteins in such a way that VPA0422 is a protein with transmembrane activity. As other authors have previously cited, proteins involved in iron uptake are possibly transmitted via horizontal gene transfer in the *Vibrio* genus. There is evidence that iron transport systems may have been acquired from other bacterial species by horizontal transfer, and this favored adaptation to humans [50]. The results presented in this study corroborate this and other works in specific situations, such as the uptake and transport of the heme group. Moreover, the presence of several genomic islands, especially PAI2 carrying these four proteins, highlights the so-called “gene promiscuity” [31,53,54] of the genus, and calls attention to the importance of horizontal gene transfer in the evolution of these pathogens, opening the possibility of further studies comparing the importance and weight of horizontal gene transfer and other point processes of genomic plasticity (such as insertions, deletions, and duplications) for the adaptation of the species to their respective hosts.

The consumption of fish and seafood is the economic basis of Asian countries, where customs and even a lack of other means have historically forced such populations to take advantage of these inputs. Among the ways to supply the local markets, aquaculture is the leading and most profitable way. However, to minimize economic losses due to bacterial infections, the exacerbated and inappropriate use of antibiotics, such as tetracycline [55,56,57,58] is routinely employed. Abusive use of antibiotics, unfortunately, leads to consequences such as pollution of the environment and also to an increase in resistant bacteria. Such a problem is already visible in several bacterial genera, and by our arguments, it is no different in *Vibrio* spp. [59,60].

In 121 genomes analyzed in the PRAP tool, 175 ARGs distributed in 17 classes were identified. Of these, 15% represent only genes associated with tetracycline resistance, an extremely high value for only one class. As a validation point of our analysis, *Vibrio cholerae* genome 2012EL 2176 showed the most ARGs in all analyses and it is precisely a multidrug-resistant strain isolated from an outbreak in Haiti [61]. Based on this, we analyzed certain factors surrounding the patterns in the distribution of ARGs. Tetracycline, in particular, is widely marketed and used in aquaculture. Taking this into consideration, the fact that all publicly available complete *V. campbellii* genomes at NCBI, as well as some isolates in seafood markets of *V. parahaemolyticus*, possess at least one tetracycline resistance gene “Tet(35)”, represents how anthropogenic action is associated with increased distribution of ARGs. Since *V. campbellii* is hardly associated with human infections, and thus, this resistance gain is not due to unsuccessful treatments from the medical clinic, which points to the resistance probably coming from the exacerbated use of tetracycline in aquaculture since it is a species found in this environment [62].

Another point that culminates in this increase in ARGs derived from human action in aquaculture is the fact that three strains of distinct species, recently isolated near Macau in China, have the highest amounts and varieties of ARGs, in addition to *V. cholerae*. The Vb1833 strain of *V. alginolyticus* isolated from shrimp, the Vb0624 strain of *V. parahaemolyticus* found in a market, and the VFN3 strain of *V. furnissii* from hospital sewage have a high resistance to several antibiotics predicted, such as sulfonamides and others. In parallel to this, when we analyzed the development of the pan-resistome of *V. cholerae*, a pathogenic representative of the genus, it is remarkable to see that it is open, with an ARG addition curve still growing, and far from a stabilization process. In other words, if for this highly evolved and well-known pathogen, there is still a tendency for the addition of ARGs, the outlook for the other species, which are poorly known in the medical clinic, may be even worse. When we compare this with the data on the three recent isolates from China, we may conclude that there is a prospect of an increase in the number of cases of vibriosis with multiple antibiotic resistance.

Since aquaculture practice is sometimes unregulated and informal, indiscriminately using antibiotics becomes a risk, not only in fish farming but also for humans. Many species will probably already come with a high burden of antibiotic resistance when this occurs because they are natural to the aquatic environment and share the environment with aquaculture. These findings draw attention because they come from species that are neglected by humans and already have a significant amount of ARGs in their genomes. With the evolutionary process and genomic plasticity, added to several global factors such as the increase in sea temperature and the dumping of plastic into the oceans, it is reasonable to think that currently non-pathogenic species may acquire the means (such as PAI2 from *V. parahaemolyticus*) to adapt and infect humans.

Finally, to combat vibriosis and cholera, we identified three drug targets for human pathogens, with very high structural quality by MHOLline2, and four drug targets for fish pathogens, with very high or high quality. This approach is cost-effective since we address several human and fish pathogens simultaneously, rather than doing drug studies separately, which would take too much time, and require many more tests for pathogens that alone do not represent how well vibriosis affects humans and fish. All drug targets have functions related to vital processes, such as energy generation in UMP kinase, making them excellent targets. In addition, we use precise and widely used tools in structural bioinformatics to determine the active site of these targets to maximize the probability that a compound, upon binding, acts by inhibiting the protein, potentially leading to bacterial death.

In the case of fish, it is known that the most common mode of drug administration is via feed. The feed, in turn, is commonly synthesized based on lipid-rich compounds for fattening animals and human consumption [63,64]. For this reason, exclusively for fish pathogens, we used 2202 of the 5008 compounds from ZINC, which presented a lipid-soluble aspect, aiming at integration and accessible administration in the feed. Thus, using AutoDock Vina, we came up with six new drug candidates, two specifics for humans and three for fish. One, in particular, was predicted for both groups identically. The natural compound “ZINC035415741” demonstrated the ability to bind to the active site of UMP kinase, making six hydrogen bonds with four residues (THR145 (3x); GLY57; GLY58; GLY18). In this sense, these new drug candidates are a way to combat pathogens for their risk and the rise in antibiotic resistance, providing new options for aquaculture and the medical clinic.

## 4. Materials and Methods

### 4.1. Data Acquisition

Our research was divided into two approaches, both in silico: comparative genomics, by addressing pan-resistome and virulence factor analysis, and drug discovery, focusing on new compounds that could be used to treat the diseases mentioned earlier. From NCBI public datasets (https://www.ncbi.nlm.nih.gov/ (accessed on 23 November 2021), we used 29 complete genome sequences of human pathogenic species (*V. alginolyticus, V. cholerae, V. parahaemolyticus, V. vulnificus, V. mimicus, V. fluvialis*), 33 fish pathogens (*V. anguillarum, V. furnissii, V. alginolyticus, V. parahaemolyticus, V. rotiferianus, V. vulnificus* biotype 2, *V. campbellii, V. harveyi*) and 27 of potential human pathogens (*V. harveyi, V. furnissii, V. campbellii, V. navarrensis, V. cincinnatiensis, V. metschnikovii, and V. metoecus*). Furthermore, no filters such as location or date were used to select the genomes. We preferentially extracted reference genomes from strains reported as pathogenic and isolated from fish and humans.

### 4.2. Adaptation and Resistance

Based on CDC epidemiological data from reliable tools such as FoodNet and CoVis, we were able to delineate 3 species groups, the HP (Human Pathogens), FP (Fish Pathogens), and RHP (Rare Human Pathogens) groups. Briefly, the species from the RHP group were selected based on epidemiological data from CoVis, selecting species with less than 5 cases per year or with 0 cases when at least 1 article citing its pathogenicity was found. We used the OrthoFinder [65] tool to analyze the genes in each group. OrthoFinder analyzes the amino acid sequence file of the entire protein set of each genome in FASTA format to perform an all-against-all comparison by BLASTp (Protein BLAST). The software uses the MCL (Markov Clustering algorithm) tool to determine genes belonging to orthologous groups.

From the orthologous genes identified by OrthoFinder, in all genomes for each cluster, we used the eggNOG-mapper tool [66] to functionally classify them based on the Cluster of Orthologous Groups (COG). Briefly, COG is a database of proteins classified by their involvement in specific processes, such as transcriptional proteins (K category) and proteins involved in ion acquisition and transport (P category), among others. To deeply analyze the differences in the K category of transcriptional processes, we used the P2RP tool [67] a web-based framework for identifying and analyzing regulatory proteins (one- and two-component systems and other transcriptional factors).

To analyze whether P category proteins, functionally annotated as associated with iron metabolism, came from genomic plasticities, such as horizontal gene transfer, we used the GIPSy tool [68]. This tool is divided into several steps, which predict genomic islands based on features commonly shared by them, such as genomic signature deviation (G + C content and codon usage), presence of transposases, flanking tRNAs, and the analysis of virulence-related content. For this, a genome from a species or strain previously classified as non-pathogenic was used. In this case, we used *Vibrio natriegens* NBRC ATCC 14,048 DSM 759 as non-pathogenic [69] and the genome of *Vibrio parahaemolyticus* strain RIMD 2,210,633 (Supplementary Table S4) as pathogenic (for human and fish). To visualize whether the proteins were in regions of genomic islands, we used the BRIG software, which performs a BLASTn and plots a circular comparison from the genomes [70].

For pan-resistome analysis and searching for ARGs, the Pan-Resistome Analysis Pipeline (PRAP) tool was used [71]. The tool uses the CARD database to search for resistance genes in each genome, traversing all possible combinations to extrapolate (by power law) the number of ARGs in the pan-resistome and core-resistome of reference species.

### 4.3. Drug Target Identification

For the discovery of new potential drug molecules against the pathogens of the *Vibrio* genus, we used the core genome of the HP and FP groups in parallel to perform subtractive genomics, using BLASTp against the host genome, an important step to avoid adverse effects of the drug, where any protein presenting homology with the human genome or the genome of the zebrafish were excluded [72]. After this, we used the SurfG+ tool [73] to check the subcellular localization of the remaining nonhost-homologous proteins, filtering only cytoplasmic proteins since these are usually involved in the basic survival processes of bacteria and essential metabolic pathways, being selected as suitable drug targets [74].

After this, we submitted the resulting proteins to the MHOLline2 web tool, a pipeline combining programs for automated protein structure prediction and detecting transmembrane regions [75]. These programs are HMMTOP (Transmembrane Helix Prediction and Protein Topology) for identifying transmembrane regions; BLAST Algorithm, performing searches in the Protein Data Bank (PDB); BATS (Automated Segmentation for Structures). HMMTOP identifies the best structural model by comparative modeling techniques, separating them according to the structural quality of the 3D construction, ranging from “very high” to “very low”; MODELLER, also performs the automation of comparative modeling of three-dimensional protein structures; and PROCHECK, which checks the stereochemical quality. Finally, MHOLline captures the structural information and returns the best 3D model. The BATS results are organized into 4 groups: G0, G1, G2, and G3. In our case, we used only the G2 proteins (E-value ≤ 10 × 10^−5^, ID ≥ 25% and LVI ≤ 0.7; LVI: Length Variation Index), which were classified with “Very High” or “High” quality. Finally, we used the DEG (Database of Essential Genes) to select only those essential proteins for bacterial survival [76]. However, this step does not exclude the previously identified targets once the proteins identified in the core genome may be recognized as essential for the genus.

### 4.4. Molecular Docking

The proteins were prepared for the molecular docking process to choose the optimal ligands. For this, we used DoGSiteScorer [77], a tool from the Protein Plus Portal Web, which can predict the active site of proteins (A binding pocket) through a difference in the Gaussian Filter to detect potential binding pockets based on the 3D protein structure. In addition, the MGLtools package (version 1.5.7) and Autodock Tool were used to prepare the target protein (charges on protein, grid box generation), where the files were converted into .pdbqt format (supported by Autodock Vina) and further used by Autodock Vina for binding analysis [78,79].

A total of 5008 drug-like molecules (natural products and their derivatives) were downloaded from the ZINC database [80] and prepared for docking analysis according to Vilela Rodrigues et al. [81]. For the human pathogen group (HP), we used all 5008 molecules, and for the fish pathogens, we used SolTranNet [82] to filter only fat-soluble compounds aiming for ease of drug delivery in animal feed. SolTranNet can predict the aqueous solubility of compounds through SMILE chemical structures. The tool is based on machine learning (ML) and uses a trained dataset, “AqSolDB,” the largest publicly available dataset defined and optimized by SolTranNet to gain speed and prediction quality. Thus, results of SMILES analysis of compounds with values of Log S > −4 are classified as soluble compounds, and Log S < −4 are predicted as insoluble compounds.

For docking analysis, we used AutoDock Vina [79]. AutoDock Vina binding affinity, number of hydrogen bonds, and active site residues were analyzed by Chimera visualization software to assess binding affinity and hydrogen bonds between ligand and protein [83] image extraction. In addition, virtual screening and top molecule (based on binding energy/affinity) identification steps were performed with the help of shell and python script, respectively (https://vina.scripps.edu/wp-content/uploads/sites/55/2020/12/vina_screen_get_top.py (accessed on 20 December 2021)). After that, the identified top molecules for each drug target (protein–ligand interaction), were analyzed together one by one to identify the best poses and binding affinity of protein–ligand interaction. The lowest energy score/binding affinity was considered [12].

## 5. Conclusions

Our research is one of the first to address the One Health perspective regarding the *Vibrio* genus. We investigate the pan-resistome of the genus, predicting the spread of ARGs and the close relationship of some resistance genes with aquaculture-associated species (such as *V. campbellii*). This possible selective pressure imposed by humans in the improper disposal of antibiotics in water streams, or aquaculture use, is reflected in the genomes of the most distinct species of the genus (from human to fish pathogens); this is a critical warning which reveals the danger of the emergence of new highly resistant bacteria that are increasingly frequent in human infections.

Another critical factor to consider is the effect of horizontal gene transfer and the acquisition of genomic islands as essential factors in adaptation to the human host. A much larger number of proteins involved in the colonization process are conserved in the human pathogen group than in others. Of these, proteins associated with iron acquisition stand out, present in putative pathogenicity islands of species such as *V. parahaemolyticus*. Thus, further studies may seek to understand the real effect of HGT on the genomic plasticity of the genus. Further studies are needed to identify proteins essential in human host colonization, among the transcriptional factors we predict as typical in HP, and to evaluate horizontal gene transfer to acquire these proteins.

**Figure 8:**
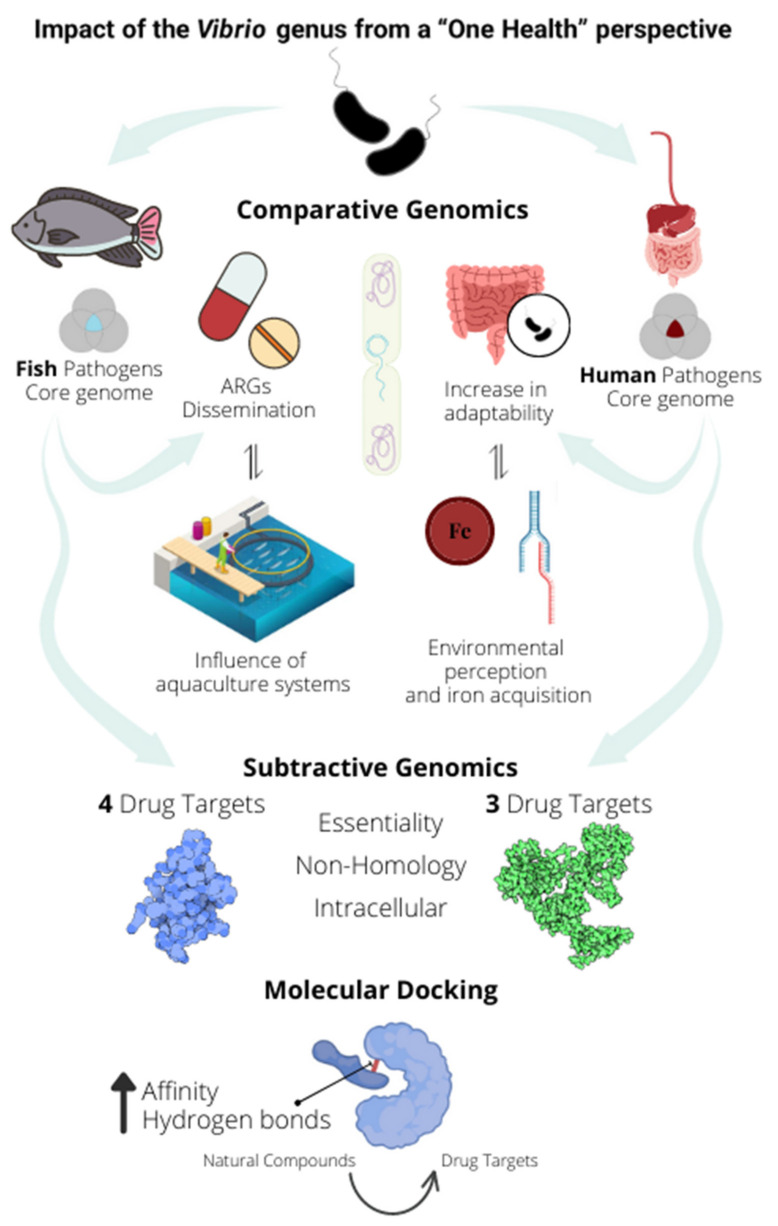
Graphical conclusion. Genomes of fish and human pathogens were used for core genome identification, and comparative analyses demonstrated the ability of pathogens to adapt to the human host linked to horizontal gene transfer and iron uptake, and the impact of selective pressure of aquaculture on the dissemination of resistance genes. Finally, a pipeline of subtractive genomics and molecular docking was performed to identify potential natural compounds capable of inhibiting the active site of the identified drug targets.

Finally, to combat this increased resistance perspective and the high dissemination of ARGs among the *Vibrio* genus, we propose three new drug targets for human pathogens, four for fish pathogens (one in common, totaling six identified proteins), and respective compounds with a high capacity to inhibit the active site of these proteins. However, more in vitro and in vivo studies are still needed to evaluate the effects of these compounds on the bacteria and the diseases above caused by them (Figure 8).

## Figures and Tables

**Table 1:** K-category (transcriptional processes) classification of the three groups, based on the P2RP tool. RR are response regulators; TR, transcriptional regulators; OCS, one-component systems and SF, sigma factors.

(COGs: K)	HP	FP	RHP
RR	12	9	7
TR	42	24	34
OCS	35	21	20
SF	7	5	5

**Table 2:** Number of genomic islands (GIs) per chromosome in the genome of *V. parahaemolyticus* RIMD, reference strain. Pathogenicity islands (PAIs) and metabolic islands (MIs), respectively.

Genome/Strain	CHR 1	CHR 2	GI’s
*V. parahaemolyticus* RIMD	11	9	PAI’s
	4	2	SI’s
	7	4	MI’s

**Table 3:** Proteins involved with iron metabolism. In the first column, IDs from the proteins found on the PAI2 of chromosome 2 of *V. parahaemolyticus*, followed by the encoding gene and the biological process involved according to the UniProt database.

Protein ID	Gene	Biological Process
WP_005491112.1	hmuV	Part of the ABC transporter complex HmuTUV involved in hemin import; Responsible for energy coupling to the transport system.
WP_005491087.1	VPA0422	Transmembrane transporter activity; Putative hemin ABC transporter, permease protein
WP_021451074.1	HutX	Heme utilization; cytosolic carrier protein
WP_005457554.1	hutZ	Heme utilization protein

**Table 4:** Information on drug targets, protein ID, products, genes, sizes (in amino acids), structural quality by MHOLline 2, and the respective biological processes retrieved from Uniprot.

	Protein ID	Product	Gene	Length (aa)	Structural Quality MHOLline	Biological Process
FP	WP_005380329.1	MULTISPECIES: UMP Kinase	pyrH	241	VERY HIGH	Catalyzes the reversible Phosphorylation of UMP to UDP. CTP biosynthesis via de novo pathway
	WP_001040192.1	MULTISPECIES: Translation initiation factor IF-1	infA	75	HIGH	Stabilizes the binding of IF-2 and IF-3 on the 30 S subunit to which N-formylmethionyl-tRNA(fMet) subsequently binds
	WP_005372901.1	MULTISPECIES: Phosphoheptose isomerase	gmhA	196	HIGH	D-glycero-D-manno-heptose 7-phosphate biosynthesis
	WP_005390081.1	MULTISPECIES: bifunctional 3-hydroxydecanoyl- ACP dehydratase/trans-2-decenoyl-ACP isomerase	fabA	172	HIGH	Fatty acid biosynthesis
HP	WP_005380329.1	MULTISPECIES: UMP kinase [*Vibrio*]	pyrH	241	VERY HIGH	Catalyzes the reversible phosphorylation of UMP to UDP. CTP biosynthesis via de novo pathway
	WP_005380392.1	MULTISPECIES: D-sedoheptulose 7-phosphate isomerase	gmhA	191	VERY HIGH	D-glycero-D-manno-heptose 7-phosphate biosynthesis
	WP_005395986.1	RNA polymerase sigma factor RpoD	rpoD	620	VERY HIGH	Promote the attachment of RNA polymerase

**Table 5:** Resumed Docking analysis. Docking results for each target in each group, the ZINC compound bound with more affinity, the binding energy and hydrogen bonds, and their residue positions accordingly.

Docking Results	Targets	ZINC Compound ID	AutoDock Vina Binding Affinity (kcal/mol)	Num. of HBonds	Residues
FP	InfA (WP_001040192.1)	ZN04235909	−7.5	1	THR54
	FabA (WP_005390081.1)	ZN04259703	−9.2	2	GLY 104, ARG 105
	GmhA (WP_005372901.1)	ZN04222852	−8.2	4	GLU156 (2×); ASP158; GLU160
	UmpK(WP_005380329.1)	ZN03541574	−10.6	6	THR145 (3×); GLY57; GLY58; GLY18
HP	UmpK (WP_005380329.1)	ZN03541574	−10.6	6	THR145 (3×); GLY57; GLY58; GLY18
	GmhA (WP_005380392.1)	ZN04259719	−8.9	7	THR120 (3x); SER55; ASN52; SER124; ASN123
	RpoD (WP_005395986.1)	ZN04236036	−10.7	3	ARG267 (2×); GLN133

Residues highlighted in red are those found in an active site.

## Supplementary Materials

The following supporting information can be downloaded at: https://www.mdpi.com/article/10.3390/antibiotics11101399/s1, Table S1: Functional annotation of pathogenic groups and the coordinates of iron-associated proteins, Table S2: Antibiotic Resistance Genes by Species, Table S3: Ligand library and receptor preparation, Table S4: Pathogens group formation and its references. References [8,12,13,14,16,17,19,20,21,22,23,25,84,85,86,87,88,89,90] are cited in the Supplementary Materials.
